# Enhancing Radiological Diagnosis: A Comprehensive Review of Image Quality Assessment and Optimization Strategies

**DOI:** 10.7759/cureus.63016

**Published:** 2024-06-24

**Authors:** Albert P Varghese, Shreya Naik, Syed Asrar Up Haq Andrabi, Anurag Luharia, Suhas Tivaskar

**Affiliations:** 1 Department of Radiology, Datta Meghe Institute of Higher Education and Research, Wardha, IND

**Keywords:** equipments, imaging modalities, general radiology, radiology artifact, metal artifacts

## Abstract

Image quality plays a pivotal role in the accurate diagnosis and effective management of diseases in radiology. This review explores the principles, methodologies, and strategies for assessing and optimizing image quality across various imaging modalities, including X-ray, computed tomography (CT), magnetic resonance imaging (MRI), ultrasound, and nuclear medicine. We discuss key factors influencing image quality, such as spatial resolution, noise, contrast, and artifacts, and highlight techniques for quality assurance, image optimization, and dose reduction in clinical practice.

## Introduction and background

The accuracy of medical pictures is crucial in radiology for both diagnosis and treatment planning. Radiological imaging modalities are constantly changing due to technological advancements, providing doctors with ever-more-detailed and educational pictures. However, these developments also bring with them new difficulties in preserving and improving picture quality while lowering related hazards like radiation exposure. A thorough awareness of the clinical demands of patients and the technical features of imaging modalities is necessary for the procedures involved in radiology assessment and optimization of picture quality. To ensure that images have enough information for radiologists to interpret them accurately, image quality assessment entails assessing several factors, including resolution, contrast, noise, and artifacts. Optimization techniques seek to reduce possible hazards, like overexposure to radiation while optimizing image quality. Radiology experts can use a variety of strategies, such as modifying imaging parameters, applying sophisticated post-processing techniques, putting quality control measures in place, and providing continuing education and training. This review delves into the key principles and strategies involved in image quality assessment and optimization in radiology. We explore the significance of image quality in diagnostic accuracy and patient care, discuss the methods used to objectively and subjectively evaluate image quality, and examine the various optimization techniques employed to enhance image quality while ensuring patient safety [1,2].

## Review

Fundamentals of image quality assessment

**Figure 1:**
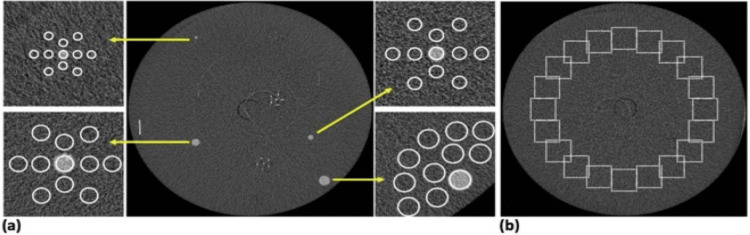
(A) Position of the region of interest for contrast-to-noise ratio (CNR) and signal-to-noise ratio (SNR) measurements. (B) Position of the region of interest for evaluating the noise power spectrum (NPS) in a uniformly distributed backdrop. This image is cited from the article, “Image quality comparison between a phase contrast synchrotron radiation breast CT and a clinical breast CT, a phantom-based study” [3].

Image quality in radiology is evaluated based on various parameters, including spatial resolution, contrast resolution, noise characteristics, and artifacts. We discuss the importance of modulation transfer function (MTF), noise power spectrum (NPS), and contrast-to-noise ratio (CNR) in quantifying image quality and their implications for clinical interpretation (Figure 1). Additionally, we explore subjective methods, such as observer performance studies and clinical feedback, in assessing image quality from a perceptual standpoint.

Spatial Resolution

The capacity of an imaging system to discriminate between two nearby features in an image is referred to as spatial resolution. Pixel size, detector geometry, and focus spot size (for X-ray imaging) are some of the parameters that affect it. A quantitative indicator of spatial resolution, the MTF, shows how effectively an imaging system replicates various spatial frequencies. Superior spatial resolution is correlated with higher MTF values.

Contrast Resolution

Contrast resolution refers to the ability of an imaging system to differentiate between tissues with different levels of contrast. It is influenced by factors such as X-ray beam energy, image acquisition parameters (e.g., kVp and mA in X-ray imaging), and image processing algorithms. The CNR quantifies the difference in signal intensity between two regions of interest relative to the noise level in the image. Higher CNR values indicate better contrast resolution.

Noise Characteristics

Noise in radiological images can arise from various sources, including electronic noise, quantum noise (associated with X-ray photon counting statistics), and patient motion. Understanding the characteristics of noise is important for optimizing imaging protocols to minimize noise levels while maintaining diagnostic image quality. The NPS provides a frequency domain representation of noise characteristics and is used to assess image quality objectively [4-6].

Artifacts

Artifacts are unwanted features in an image that result from limitations or errors in the imaging process. They can degrade image quality and confound diagnostic interpretation. Common artifacts in radiology include motion artifacts, beam-hardening artifacts (in CT imaging), metal artifacts, and aliasing artifacts (in MRI). Identifying the underlying causes of artifacts is crucial for implementing corrective measures and optimizing imaging protocols.

Subjective vs. Objective Assessment

Image quality can be assessed subjectively through visual inspection by radiologists or objectively using quantitative metrics. Subjective assessment relies on the radiologist's experience and perception to evaluate image quality, while objective assessment involves the use of numerical measures, such as MTF, CNR, and NPS, to quantify specific aspects of image quality. Both subjective and objective assessments complement each other and are essential for comprehensive image quality evaluation.

Quality Assurance and Optimization

Quality assurance programs are established in radiology departments to ensure consistent image quality and compliance with regulatory standards. These programs include routine equipment maintenance, calibration, and performance testing to monitor and optimize imaging system performance. Technologist training and ongoing education are also essential components of quality assurance efforts to maintain high standards of image quality.

Optimization Techniques in Radiological Imaging

Optimizing image quality involves balancing competing factors, such as radiation dose, contrast enhancement, and temporal resolution, to achieve diagnostically acceptable images. We review optimization strategies tailored to different imaging modalities, including parameter selection, protocol optimization, iterative reconstruction algorithms, and postprocessing techniques. Special emphasis is placed on dose reduction techniques in X-ray and CT imaging, including automatic exposure control (AEC), tube current modulation, and iterative reconstruction methods [7-9].

X-ray imaging

Parameter Selection

Optimization in X-ray imaging involves selecting appropriate exposure parameters, such as tube voltage (kVp), tube current (mA), and exposure time (mAs), to achieve adequate image contrast and resolution while minimizing radiation dose.

Automatic Exposure Control

AEC systems adjust radiation exposure based on patient anatomy and tissue density, ensuring consistent image quality while reducing unnecessary radiation exposure.

Iterative Reconstruction

Iterative reconstruction algorithms improve image quality and reduce noise compared to traditional filtered-back projection (FBP) methods, allowing for dose reduction without compromising diagnostic accuracy.

Computed tomography

Protocol Optimization

Optimizing CT protocols involves selecting appropriate acquisition parameters (kVp, mA, pitch, and rotation time) based on patient characteristics and clinical indications to balance image quality and radiation dose.

Tube Current Modulation

Tube current modulation techniques vary tube current during scanning based on patient attenuation, reducing dose while maintaining image quality.

Iterative Reconstruction

Iterative reconstruction algorithms in CT improve image quality, reduce noise, and enable dose reduction by using statistical models to reconstruct images from raw data.

Magnetic resonance imaging

Sequence Optimization

Optimizing MRI sequences involves selecting appropriate pulse sequences, echo times (TE), repetition times (TR), and flip angles to achieve optimal tissue contrast and spatial resolution.

Parallel Imaging

Parallel imaging techniques, such as sensitivity encoding (SENSE) and generalized autocalibrating partially parallel acquisitions (GRAPPA), accelerate image acquisition, reducing scan time and motion artifacts.

Motion Correction

Motion correction techniques, such as prospective motion correction and motion-compensated reconstruction, minimize artifacts caused by patient motion during MRI scanning, improving image quality [10-12].

Ultrasound imaging

Transducer Selection

Choosing the appropriate ultrasound transducer frequency and configuration based on the depth of the target structure and desired resolution optimizes image quality.

Image Optimization Settings

Adjusting imaging parameters such as gain, depth, focus, and dynamic range optimizes ultrasound images for specific clinical applications and patient characteristics.

Contrast Enhancement Techniques

Contrast-enhanced ultrasound (CEUS) techniques improve the visualization of vascular structures and enhance image contrast for better diagnostic accuracy.

Nuclear medicine imaging

Radiopharmaceutical Optimization

Selecting appropriate radiopharmaceuticals and administration protocols optimizes image quality and tracer uptake, ensuring accurate diagnosis and quantification of physiological processes.

Image Reconstruction Techniques

Iterative reconstruction algorithms in nuclear medicine imaging improve image quality, reduce noise, and enhance lesion detectability, allowing for dose reduction while maintaining diagnostic accuracy.

Motion Correction

Motion correction techniques, such as respiratory gating and motion tracking, minimize artifacts caused by patient motion during image acquisition, improving spatial resolution and image quality.

Artifacts and error correction

Artifacts can degrade image quality and obscure diagnostic information in radiological images. We categorize common artifacts based on their underlying causes, such as patient motion, equipment malfunctions, and image reconstruction errors. We discuss strategies for artifact prevention and correction, including motion compensation algorithms, metal artifact reduction techniques (MARTs), and software-based postprocessing filters.

Types of artifacts

Motion Artifacts

Motion artifacts result from patient movement during image acquisition and can manifest as blurring or ghosting of anatomical structures. Techniques, such as immobilization devices, breath-holding instructions, and motion correction algorithms, can mitigate motion artifacts.

Beam-Hardening Artifacts

Beam-hardening artifacts occur due to differential absorption of X-ray photons by tissues of varying densities, leading to streaking artifacts in CT images. Correction methods include using beam-hardening correction algorithms and acquiring images with higher tube voltages.

Metal Artifacts

Metal implants or objects within the patient can cause streaking or shading artifacts due to photon starvation or scatter. MARTs, such as iterative reconstruction algorithms and dual-energy CT, can reduce the impact of metal artifacts.

Aliasing Artifacts

Aliasing artifacts occur when high-frequency signals are incorrectly represented as low-frequency signals, leading to pixelation or wraparound artifacts. Increasing the field of view or spatial resolution, as well as using antialiasing filters, can mitigate aliasing artifacts.

Slice Thickness Artifacts (MRI)

Slice thickness artifacts in MRI result from partial volume effects when the slice thickness is greater than the object size. Using thinner slice thickness, increasing the matrix size, or applying interpolation techniques can reduce slice thickness artifacts.

Gradient Nonlinearity Artifacts (MRI)

Gradient nonlinearity artifacts occur due to spatial distortion caused by nonuniform magnetic field gradients. Correction methods involve calibrating gradient field maps and applying distortion correction algorithms during image reconstruction [13-15].

Strategies for artifact correction

Prevention

Minimizing artifacts begins with proper patient preparation, positioning, and immobilization to reduce motion-related artifacts. Optimizing imaging parameters, such as adjusting scan protocols and choosing appropriate acquisition techniques, can also prevent artifacts.

Image Reconstruction Algorithms

Advanced reconstruction algorithms, such as iterative reconstruction in CT and parallel imaging in MRI, can reduce noise and artifacts while preserving image quality. These algorithms use statistical models and regularization techniques to improve image fidelity.

Postprocessing Techniques

Postprocessing software tools, such as artifact reduction filters and image fusion algorithms, can be employed to correct artifacts retrospectively. These techniques enhance image quality and improve diagnostic accuracy by removing or minimizing artifact-induced distortions.

Artifact-Specific Correction Methods

For specific artifacts, like metal artifacts in CT, specialized correction methods, such as MARTs, are available. These techniques involve sophisticated algorithms that model and compensate for the effects of metal objects on image reconstruction.

Quality assurance and regulatory compliance

Quality assurance programs are essential for ensuring consistent image quality and patient safety in radiology departments. We outline the components of a comprehensive quality assurance program, including equipment calibration, performance testing, and compliance with regulatory standards such as the American College of Radiology (ACR) accreditation requirements. We also highlight the role of technologist training and ongoing education in maintaining high standards of image quality.

Components of quality assurance

Equipment Performance Testing

Regular performance testing of imaging equipment, including X-ray machines, CT scanners, MRI systems, and ultrasound machines, ensures proper functionality and adherence to manufacturer specifications. Tests may include checks of image quality parameters, radiation output, and geometric accuracy.

Image Quality Assurance

Continuous monitoring of image quality parameters, such as spatial resolution, contrast resolution, and noise characteristics, is essential for maintaining diagnostic image quality. Quality control tests, such as phantom imaging and analysis, are performed regularly to assess image quality and detect any deviations from established standards.

Radiation Dose Monitoring

Monitoring patient radiation dose exposure is crucial for optimizing imaging protocols and minimizing radiation risks. Dose tracking software and dose monitoring systems enable real-time monitoring of patient dose metrics, facilitating dose optimization and dose reduction strategies.

Personnel Training and Certification

Ongoing training and education programs ensure that imaging technologists and radiologists possess the necessary skills and knowledge to perform imaging procedures safely and accurately. Certification programs, such as those offered by professional organizations like the American Registry of Radiologic Technologists (ARRT), validate competency and adherence to professional standards.

Regulatory requirements

Accreditation Organizations

Accreditation bodies, such as the ACR and the Intersocietal Accreditation Commission (IAC), accredit imaging facilities based on compliance with established standards and guidelines. Accreditation demonstrates commitment to quality and patient safety and may be required for reimbursement and licensure.

State Regulations

Some states have additional regulations governing radiological imaging practices, including requirements for facility licensure, personnel certification, and radiation safety. State regulatory agencies oversee compliance with these regulations and may conduct inspections to ensure adherence to standards [16-20].

Strategies for Compliance

Establishment of Quality Assurance Programs

Radiology departments and imaging facilities should develop comprehensive quality assurance programs that encompass equipment testing, image quality assessment, radiation dose monitoring, and personnel training. These programs should be regularly reviewed and updated to reflect changes in technology and regulatory requirements.

Adherence to Accreditation Standards

Facilities seeking accreditation should familiarize themselves with accreditation standards and guidelines established by accrediting bodies such as the ACR and IAC. Compliance with these standards demonstrates a commitment to quality and patient safety and facilitates reimbursement and recognition.

Continuous Quality Improvement

Continuous quality improvement initiatives involve ongoing monitoring, evaluation, and optimization of imaging practices to enhance quality and efficiency. Regular performance audits, clinical feedback mechanisms, and process improvement initiatives contribute to a culture of continuous improvement and excellence in patient care.

Emerging trends and future directions

Advancements in technology, such as artificial intelligence (AI), deep learning, and quantitative imaging, hold promise for further improving image quality and diagnostic accuracy in radiology. We discuss the potential applications of AI algorithms in image reconstruction, artifact detection, and image enhancement, as well as the integration of quantitative imaging biomarkers into routine clinical practice for personalized patient care.

Artificial Intelligence and Machine Learning

AI and machine learning are poised to transform radiological imaging by automating image analysis, enhancing diagnostic accuracy, and enabling personalized medicine. AI algorithms can analyze large datasets, detect subtle abnormalities, and predict patient outcomes, leading to more efficient and precise diagnoses. Future developments may include AI-driven image reconstruction, automated lesion detection, and the integration of AI tools into the radiology workflow.

Quantitative Imaging and Radiomics

Quantitative imaging techniques and radiomics analysis extract quantitative data from medical images to characterize tissue properties and predict disease prognosis. Radiomics models combine imaging features with clinical and genomic data to improve disease diagnosis, treatment planning, and response assessment. The integration of radiomics into routine clinical practice holds promise for personalized medicine and precision oncology.

Molecular Imaging and Therapeutic

Molecular imaging modalities such as positron emission tomography (PET) and single-photon emission computed tomography (SPECT) enable non-invasive visualization and quantification of molecular processes in vivo. Theragnostic, which combines diagnostic imaging with targeted therapy, offers personalized treatment options based on individual patient characteristics and disease biology. Advancements in radiopharmaceutical development and molecular imaging techniques are driving the growth of therapeutics in oncology and beyond.

Hybrid Imaging Modalities

Hybrid imaging combines two or more imaging modalities, such as PET/CT and PET/MRI, to provide complementary anatomical and functional information in a single examination. Hybrid imaging enhances diagnostic accuracy, improves lesion detection, and facilitates treatment planning by integrating structural and molecular imaging data. Future developments may include multimodal imaging platforms with integrated AI capabilities for comprehensive disease evaluation.

Interventional radiology and image-guided therapies

Interventional radiology (IR) techniques utilize imaging guidance, such as fluoroscopy, ultrasound, and CT, to perform minimally invasive procedures for diagnosis and treatment. Emerging trends in IR include the development of image-guided therapies, targeted drug delivery systems, and minimally invasive surgical techniques. Real-time imaging and navigation technologies enhance procedural precision and patient safety in IR procedures.

Point-of-Care and Portable Imaging Devices

Advancements in portable imaging devices, such as handheld ultrasound and mobile X-ray units, enable point-of-care imaging in diverse clinical settings, including emergency departments, intensive care units, and remote locations. Portable imaging devices improve accessibility to diagnostic imaging services, expedite patient care, and facilitate rapid clinical decision-making in resource-limited settings [21-23].

Discussion

Radiological diagnosis heavily relies on the quality of the images produced by various imaging modalities. The importance of image quality in radiology cannot be overstated, as it directly impacts diagnostic accuracy and, consequently, patient outcomes. This discussion explores the critical aspects of image quality assessment and optimization in radiology, addressing current challenges and potential advancements. In the last few years, technological developments in the surgical field have been rapid and are continuously evolving. One of the most revolutionary breakthroughs was the introduction of the Internet of Things (IoT) concept within the medical practice [24].

Significance of Image Quality in Radiology

High-quality images are essential for accurate diagnosis, treatment planning, and follow-up in various medical conditions. Poor image quality can lead to misdiagnoses, overlooked pathologies, and unnecessary repeat examinations, which not only increase healthcare costs but also expose patients to additional radiation. Therefore, ensuring optimal image quality is a fundamental goal in radiological practices.

Image Quality Assessment

Image quality assessment involves both subjective and objective methods. Subjective assessment relies on the radiologist's perception of image clarity, contrast, and detail. However, subjective evaluations can be inconsistent due to human variability. Objective assessment, on the other hand, employs quantitative metrics, such as signal-to-noise ratio (SNR), CNR, and spatial resolution, to provide a more standardized evaluation. Advances in machine learning and AI offer promising tools for automating and enhancing objective image quality assessments, potentially leading to more consistent and reproducible results.

Optimization Techniques

Optimizing image quality involves adjusting various parameters within imaging systems to achieve the best possible images while minimizing patient exposure to radiation. Key optimization strategies include the following:

Adjusting imaging parameters: Modifying parameters, such as tube voltage, current, and exposure time, can significantly impact image quality. Tailoring these settings to individual patient characteristics and clinical needs is crucial.

Postprocessing algorithms: Advanced image reconstruction and postprocessing algorithms can enhance image quality by reducing noise and improving contrast. Techniques, such as iterative reconstruction and deep learning-based algorithms, have shown promise in producing high-quality images at lower radiation doses.

Quality control programs: Implementing rigorous quality control programs ensures that imaging equipment operates at optimal performance levels. Regular calibration and maintenance of equipment are essential components of these programs.

Training and education: Continuous education and training of radiology personnel on the latest techniques and best practices in image acquisition and processing are vital for maintaining high standards of image quality [25-28].

Challenges and future directions

Despite significant advancements, several challenges remain in the pursuit of optimal image quality in radiology.

Balancing Image Quality and Radiation Dose

Striking the right balance between achieving high image quality and minimizing radiation exposure remains a complex challenge, particularly in pediatric and high-risk populations.

Technological Variability

The rapid evolution of imaging technologies can lead to variability in image quality. Ensuring consistent quality across different devices and manufacturers requires standardized protocols and interoperability.

Data Integration and AI Implementation

Integrating AI tools into clinical workflows for image quality assessment and optimization poses challenges related to data privacy, interoperability, and clinician acceptance. Moreover, the need for large annotated datasets to train AI models is a significant barrier. Looking ahead, the integration of AI and machine learning in radiology holds great potential for transforming image quality assessment and optimization. AI can provide real-time feedback during image acquisition, predict and correct artifacts, and personalize imaging protocols based on patient-specific factors. Continued research and collaboration among radiologists, medical physicists, and AI experts will be essential to harnessing these technologies effectively [29-31].

## Conclusions

In radiology, obtaining precise diagnoses and enhancing patient outcomes depend heavily on optimizing picture quality. Radiologists may improve their practices' diagnostic confidence and efficiency, which will eventually benefit the patients they serve, by comprehending the fundamentals of picture quality evaluation and putting optimization tactics into practice.

Achieving excellent image quality requires using cutting-edge imaging technology such as sophisticated image processing algorithms and high-resolution digital detectors. By understanding the principles of image quality assessment and implementing optimization strategies, radiologists can enhance their practices' diagnostic confidence and efficiency, ultimately benefiting the patients they serve. Enhancing image quality in radiology is a multifaceted endeavor that requires a combination of advanced technology, rigorous quality control, and continuous education. By addressing the existing challenges and embracing innovative solutions, the radiology community can significantly improve diagnostic accuracy and patient care.
